# Pharmacological and Therapeutic Properties of the Caucasian Whortleberry (*Vaccinium arctostaphylos* L.): An Overview of the New Findings

**DOI:** 10.1002/fsn3.70702

**Published:** 2025-08-07

**Authors:** Hesam Bighamanganeh, Maryam Salabati, Abbas Khalilpour, Mohsen Akbaribazm, Nabi Akbarnezhad

**Affiliations:** ^1^ Department of Basic Medical Sciences Khoy University of Medical Sciences Khoy Iran; ^2^ Department of Operating Room Khoy University of Medical Sciences Khoy Iran; ^3^ Department of Nursing Qazvin University of Medical Sciences Qazvin Iran

**Keywords:** anthocyanin, antidiabetic, antioxidant, medicinal plant, *V. arctostaphylos*

## Abstract

*Vaccinium arctostaphylos* L. (*V. arctostaphylos*) is an herb renowned in traditional medicine for its diverse therapeutic applications, including the treatment of skin ulcers, hyperlipidemia, liver diseases, and gastrointestinal issues. In this study, we conducted a comprehensive review of the botanical, phytochemical, and therapeutic effects of *V. arctostaphylos* to provide researchers with insights into its potential use in treating various disorders. Employing systematic review methods with keywords such as “*Vaccinium arctostaphylos*,” “Clinical trial,” “Therapeutic properties,” and “Animal studies,” we analyzed 225 articles extracted from databases like PubMed, ScienceDirect, SID, and Google without time constraints. Following rigorous title and abstract screening, 60 articles were selected for analysis. Volatile compound analysis revealed a rich array present in both the fruit and leaves, suggesting applications across diverse fields. Nutritional analysis underscored significant protein, sugar, fat, and fiber contents, alongside essential minerals and vitamins. Pharmacologically, *V. arctostaphylos* demonstrated a range of beneficial properties, including antihypertensive, anti‐obesity, antihyperlipidemic, anti‐inflammatory, antioxidant, antiplatelet, anti‐cancer, and antibacterial effects. Clinical trials supported its efficacy in reducing blood pressure, improving lipid profiles, and managing diabetes. Mechanistic insights highlighted its action through various pathways, including modulation of glucose metabolism, inhibition of inflammatory mediators, and enhancement of antioxidant defenses. Furthermore, *V. arctostaphylos* showed promise in addressing cardiovascular health, diabetes, male reproductive system issues, and neurological disorders. Its antimicrobial potential against various pathogens suggests utility in infection management. This comprehensive overview underscores the multifaceted therapeutic potential of *V. arctostaphylos*, affirming its importance in both medicinal and nutritional contexts.

## Graphical abstract

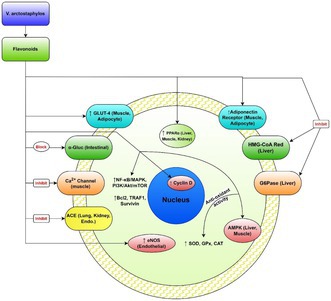

## 1 Introduction

Medicinal plants play a pivotal role in traditional medicine across diverse nations and cultures, with some species being widely distributed while others are native to specific regions and utilized for treating various ailments. In modern medicine, extracts, active ingredients, and purified plant compounds are commonly incorporated into treatment processes alongside contemporary methods (Fitzgerald et al. 2020). *Vaccinium arctostaphylos* L. (*V. arctostaphylos*), also known as Qaraqat, vaccinium, Siah Gileh, Arctic raspberry, or Arctic bramble, is a flowering plant in the Ericaceae family native to the Arctic and subarctic regions of Europe, Asia, and North America (Figure 2A–D). It is a deciduous shrub with small, alternate leaves and bell‐shaped white to pinkish flowers that grow in clusters, producing small round berries typically red or dark purple when ripe (Figure 3) (Bussmann et al. 2020). This species thrives in acidic, well‐drained soils found in arctic and subarctic habitats such as tundra, boreal forests, and mountainous areas (Nickavar et al. 2002).

*V. arctostaphylos* contains a range of bioactive phytochemicals, including flavonoids, isoflavonoids, and anthocyanins. Key anthocyanins like delphinidin, petunidin, and malvidin have been identified in the fruit using liquid chromatography‐electrospray ionization‐mass spectrometry (LC‐ESI‐MS), with these compounds linked to sugars such as arabinose and glucose (Mollaamin et al. 2021; Karabulut and Celik 2013). Studies show high levels of anthocyanins (1420 mg/100 g dry weight), polyphenols (11.539–20.742 mg GAE/g), and flavonoids (1.182–2.676 mg QE/g) in the plant's fruit and leaves, which include kaempferol glycosides, quercetin, and myricetin (Ehlenfeldt and Ballington 2012; Nickavar and Amin 2004; Cabrita and Andersen 1999). Additionally, phenolic antioxidants like pterostilbene, resveratrol, and various hydroxycinnamic acids contribute to the plant's medicinal properties (LAtti et al. 2009). Volatile compounds in *V. arctostaphylos* have been analyzed using GC/MS, revealing 26 key compounds in the fruit, including alpha‐terpineol (14.99%) and linalool (13.7%) (Musavi et al. 2016; Nickavar et al. 2002) (Table 1). The leaves contain 338 volatile compounds, including terpenoids and fatty acids, with α‐terpineol (17%) being the most abundant (Radulović et al. 2010). Additionally, fructose, glucose, sucrose, and volatile acids such as malic, quinic, and citric acids are dominant in the fruit. Nutritionally, the fruit contains 15.5% protein, 30.6% sugars, and 22.3% fiber, while the leaves hold 11.3% protein, 20.7% sugars, and 42.4% fiber (Ayaz et al. 2001). Both leaves and fruit are rich in essential minerals like calcium, magnesium, potassium, and iron, along with vitamins A, K, C, and D, further supporting the plant's therapeutic potential (Sedaghathoor et al. 2006). Traditionally, indigenous communities have used *V. arctostaphylos* for both food and medicinal purposes, consuming its berries and leaves. The berries contain phytochemicals like anthocyanins and flavonoids, contributing to their antioxidant and anti‐inflammatory properties (Ockun et al. 2022).

**TABLE 1:** The predominant anthocyanins, flavonols and polyphenols of the *V. arctostaphylos* plant.

Phytocompounds	Major compounds	Structural configuration			Chemical structure
Anthocyanins	Anthocyanin derivatives	R_1_	R_2_		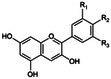
	Delphinidin	OH	OH		
	Cyanidin	OH	H		
	Petunidin	OCH_3_	OH		
	Peonidin	OCH_3_	H		
	Malvidin	OCH_3_	OCH_3_		
Flavonols	Flavonoids	R_1_	R_2_		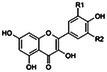
	Kaempferol	H	H		
	Quercetin	OH	H		
	Myricetin	OH	OH		
Polyphenolic compounds	Cinnamic acid derivative	R4	R5	R6	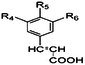
	m‐Coumaric acid	OH	H	H	
	p‐Coumaric acid	H	OH	H	
	Caffeic acid	OH	OH	H	
	Ferulic acid	OCH_3_	OH	H	
	Sinapinic acid	OCH_3_	OH	OCH_3_	
Benzoic acid derivatives	Benzoic acid derivatives	R_1_	R_2_	R_3_	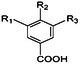
	p‐hydroxybenzoic acid	H	OH	H	
	Protocatechuic acid	OH	OH	H	
	Gallic acid	OH	OH	OH	
	Syringic acid	OCH_3_	OH	OCH_3_	
	3,5‐Dihydroxybenzoic acid	OH	H	OH	

In this review study, we tried to have a comprehensive review of the botanical, phytochemical, and therapeutic effects of the *Vaccinium arctostaphylos* L. (*V. arctostaphylos*) to provide a way for researchers to use this plant in the treatment of various disorders and future studies.

## 2 Materials and Methods

### 2.1 Search Strategy and Study Selection

**FIGURE 1:**
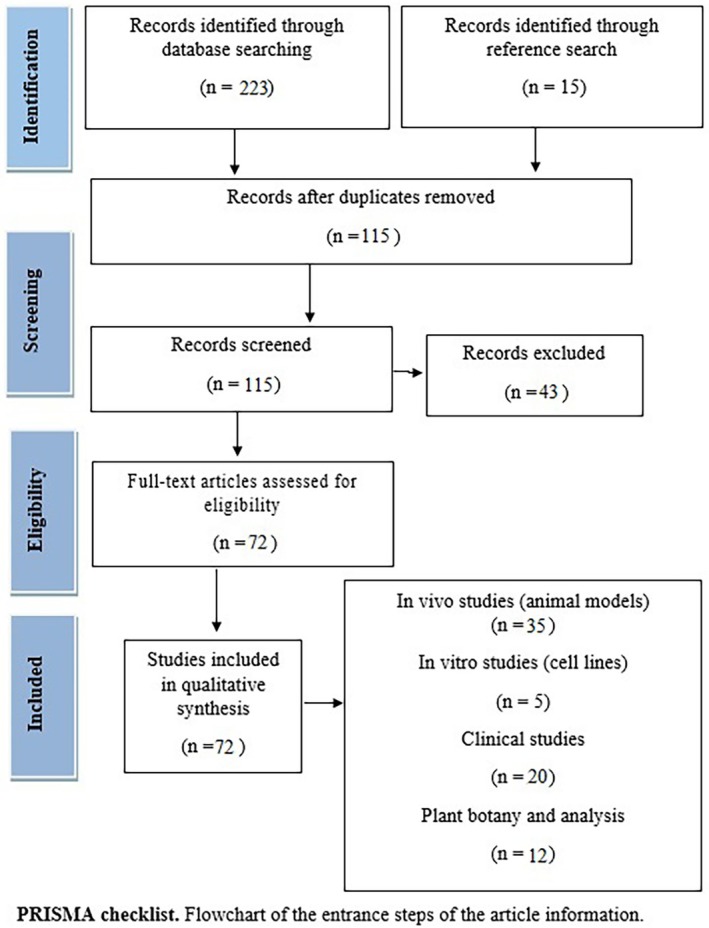
Flowchart of literature search and selection process.

For this study, a review was conducted on 225 articles gathered from various databases including the Cochran library, Iranmedex, Wiley Online Library, WHO, Science Direct, Scientific Information Database (SID), PubMed, and Google Scholar search engine. The search was not time‐limited and utilized MeSH keywords such as Arctostaphylos, Ethnobotany, Flavonoids, Pharmacognosy, Protective agents, Anti‐Inflammatory agents, Anticancer agents, Cardiovascular agents, Gas chromatography–mass spectrometry, Antioxidants, Endocrine system diseases, Hyperlipidemia, Urogenital system, Hypercholesterolemia, Diabetes mellitus, Glucose metabolism disorders, and Edible plants. After removing duplicate studies, 61 articles relevant to the study were selected (Figure 1). The review primarily discusses the botany, phytochemistry, therapeutic effects, and antioxidant and anti‐inflammatory properties of *V. arctostaphylos* in various in vitro and in vivo (animal and human) models. Most articles examined its antidiabetic effects in animal models, followed by studies on phytochemistry and its antioxidant effects. Analysis of the types of studies conducted over a 30‐year period reveals that the majority were carried out between 2019 and 2024. Based on the study's findings, further research is recommended on the plant's antitumor effects and its potential role in infertility treatment in women, considering its high antioxidant properties and content of polyphenolic‐anthocyanin compounds, total phenolic content (TPC), and total flavonoid content (TFC) in extracts.

## 3 Results & Discussion

### 3.1 Botany and Edible Uses

**FIGURE 2:**
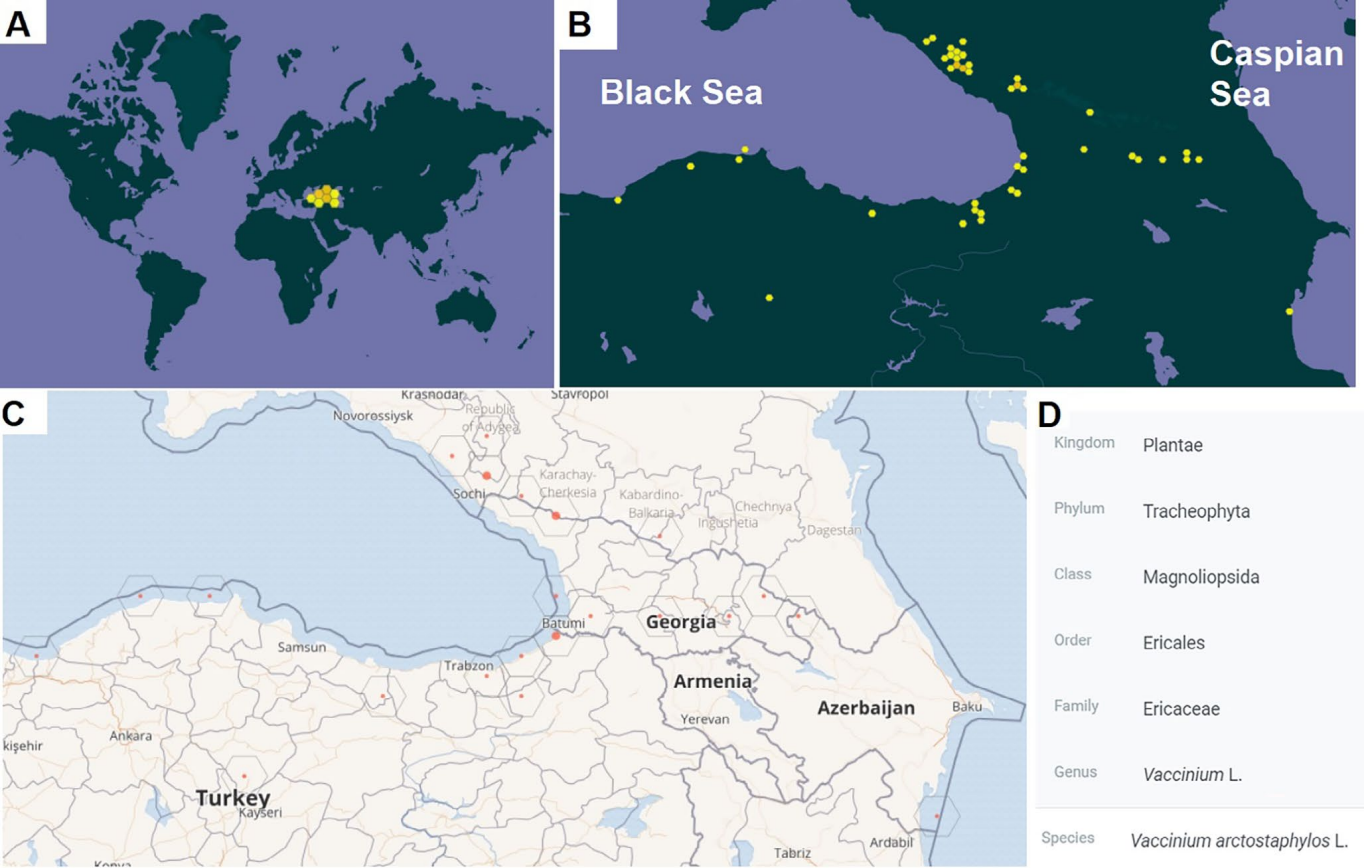
(A, B) Geographical distribution of *V. arctostaphylos* growth in southern and eastern Europe as well as northwestern Asia based on iNaturalist research‐grade observations. (C) *V. arctostaphylos* growth centers. The main cities are marked with a hexagonal frame and the amount of aggregation of this plant with the help of red color density. (D) Scientific classification of *V. arctostaphylos*.

**FIGURE 3:**
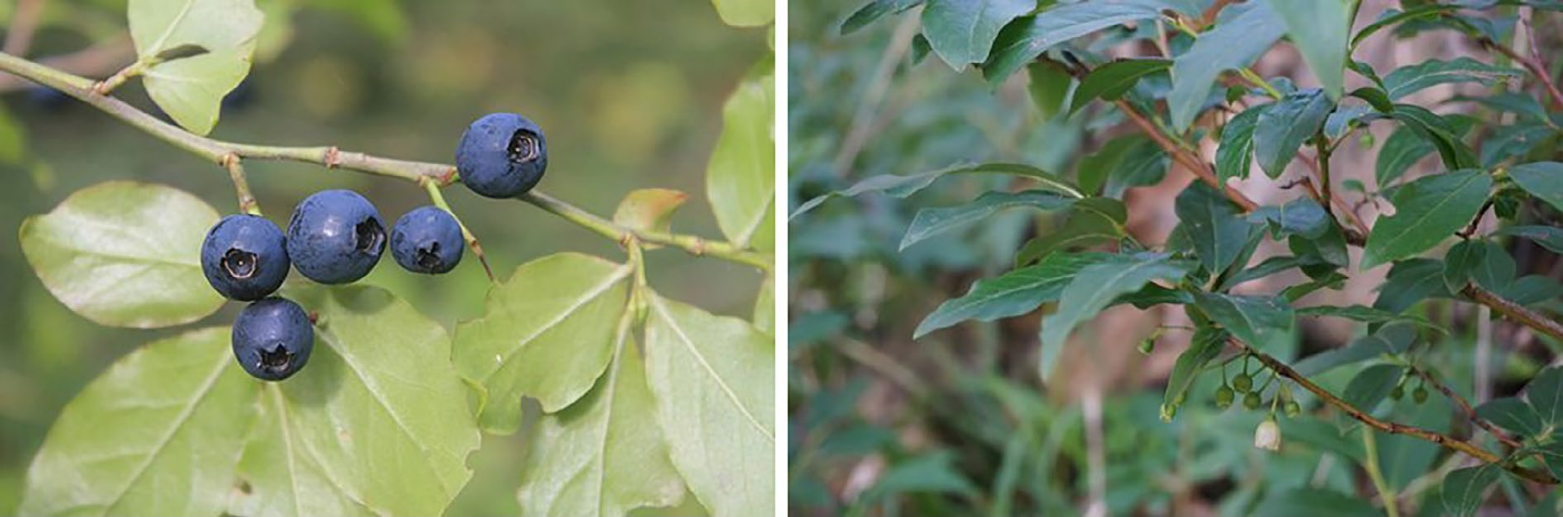
*V. arctostaphylos* plant and its fruits observed in the Russian Federation (https://www.inaturalist.org/observations/54445592).

The edible plant *V. arctostaphylos* is native to Eastern Europe, particularly the region between the Black Sea and the Caspian Sea, encompassing countries like the Russian Federation, Turkey, Georgia, Bulgaria, Azerbaijan, Armenia, and Western Asia, including northwestern Iran (Figure 2A–C). Among the 28 species of the Vaccinium genus, seven are recognized for their medicinal properties, including *Vaccinium angustifolium* L., *Vaccinium arboretum* L., *Vaccinium austral* L., *Vaccinium bracteatum* L., *Vaccinium caespitosum*, *Vaccinium corymbosum* L., and *V. arctostaphylos* (Song and Hancock 2011). Known locally as Qaraqat, vaccinium, or Siah Gileh in northern Iran, *V. arctostaphylos* is characterized by short, perennial, tetraploid shrubs reaching approximately 1.5 to 2.5 m in height, with numerous downward branches. Typically found at altitudes between 900 to 1250 m above sea level, its fruit is purple‐black to black (Mollaamin et al. 2021). Belonging to the Ericaceae family, *V. arctostaphylos* is hermaphroditic, exhibiting both male and female organs (Figure 2D). The plant flowers from early May to July, with fruit ripening in late September. Its leaves, almost lacking petioles or with short ones, are toothed, slender, oval, or elongated ovate, measuring 1–3 cm long, with smooth edges and light green coloration. *V. arctostaphylos* thrives in moist to semi‐moist soils with acidic to very acidic pH levels, primarily in forest‐mountain habitats characterized by high humidity and varying light conditions. Its pear‐shaped fruit, about 8–10 mm in length, possesses acidic properties and is utilized for its healing attributes (Figure 3) (Ehlenfeldt and Ballington 2012). In regions along the Black Sea and the Caspian Sea, particularly in northwestern Iran and eastern Turkey, dried leaves of *V. arctostaphylos* serve as a tea substitute and are added to local cuisines, pickles, and soups. Traditional medicinal practices utilize different parts of the plant, particularly the dried fruit extract, for managing conditions such as blood sugar, hyperlipidemia, hypertension, cardiovascular and gastrointestinal disorders, kidney and bladder stones, and male infertility (Sultana et al. 2020; Soltani et al. 2014).

### 3.2 Phytochemical Constituents

#### 3.2.1 Flavonoids, Isoflavonoids, and Anthocyanins of *V. arctostaphylos*

LC‐ESI/MS analysis of *V. arctostaphylos* fruit extracts confirmed the presence of anthocyanin compounds, including delphinidin, petunidin, and malvidin (Nickavar and Amin 2004). Another study on the fruit of this plant, employing a combination of high‐performance liquid chromatography (HPLC) and nuclear magnetic resonance techniques, revealed that aglycones cyanidin, delphinidin, malvidin, petunidin, and peonidin are conjugated with sugars such as arabinose, glucose, and galactose (Cabrita and Andersen 1999). Furthermore, an analysis based on HPLC combined with diode array (DAD) and electrospray ionization mass spectrometric (ESI‐MS) methods showed that the mean total anthocyanin content in this plant was 1420 mg/100 g dry weight, with delphinidin (41%), petunidin (19%), and malvidin (19%) being the major anthocyanins, consistent with other studies (LAtti et al. 2009). *V. arctostaphylos* exhibits a high content of polyphenols (11.539–20.742 mg GAE/g dry sample) and flavonoids (1.182–2.676 mg QE/g dry sample). Major flavonoid compounds identified in the fruit and leaves of this plant include kaempferol glycosides, myricetin‐3‐arabinoside, quercetin‐3‐arabinoside, and quercetin‐7‐glucoside, as determined by LC‐ESI/MS analysis (Saral et al. 2015; Linderborg et al. 2011). Additionally, Braga et al. (2013) identified flavonoid compounds such as myricetin, kaempferol, and quercetin, as well as phenol‐like antioxidants pterostilbene and resveratrol, hydroxycinnamic acids (ferulic, caffeic, and coumaric acid), and hydroxybenzoic acids (procatechuic and gallic acid) in leaf and fruit extracts of this plant (Braga et al. 2013; Musavi et al. 2016) (Table 1).

#### 3.2.2 Volatile Compounds of *V. arctostaphylos*

A study conducted by Nickavar et al. (2002) employing GC/MS analysis on the fruit of *V. arctostaphylos* revealed the presence of 26 volatile compounds, with alpha‐terpineol (14.99%) and linalool (13.7%) identified as the major volatile compounds. Among the volatile compounds (> 1%) identified were cis‐linalool oxide (3.19%), trans‐linalool oxide (2.31%), n‐nonanal (1.51%), ho‐trienol (2.10%), safranal (1.55%), beta‐citronellol (3.40%), trans‐geraniol (6.27%), vitispirane (2.8%), thymol (6.54%), carvacrol (1.84%), α‐Ionone (1.19%), geranyl acetone (1.16%), β‐Ionone (7.53%), dihydroactinidiolide (2.33%), nonadecane (1.16%), and eicosane (1.73%) (Nickavar et al. 2002). Additionally, GC/MS analysis of the essential oils hydro‐distilled from dry leaves of *V. arctostaphylos* revealed the presence of 338 volatile compounds, including terpenoids, fatty acids, and carotenoid‐derived compounds. The major volatile compound identified in the leaves was α‐terpineol (17%). Among other volatile compounds (> 1%) detected were nonacosane (2%), 1‐octacosene (1.6%), heptacosane (2.9%), 1‐hexacosene (1.1%), pentacosane (6.4%), tetracosane (1.2%), 1‐tetracosene (1.4%), tricosane (2.1%), 13‐epi‐manool oxide (2%), manoyl oxide (1.4%), hexahydrofarnesyl acetone (1.7%), germacrone (1.1%), valerianol (1.7%), 10‐epi‐γ‐eudesmol (3%), α‐agarofuran (1.8%), α‐farnesene (4.9%), β‐Ionone (1.1%), α‐humulene (1.1%), geraniol (1.5%), and linalool (4.7%) (Radulović et al. 2010). Furthermore, a study utilizing gas liquid chromatography mass spectrometry (GLC‐MS) and verified by gas liquid chromatography (GLC) on the sugar and acidic volatile compounds of the fruit of *V. arctostaphylos* identified fructose, glucose, and sucrose as the predominant sugar compounds, while volatile acids were dominated by malic acid, quinic acid, and citric acid (Ayaz et al. 2001).

#### 3.2.3 Other Compounds (Minerals, Vitamins and Etc.) of *V. arctostaphylos*

The fruit of *V. arctostaphylos* comprises 15.5% protein, 30.6% sugars, 2% soluble solids, and 1.5% total fat, while its leaves contain 11.3% protein, 20.7% sugars, 4% soluble solids, and 1% total fat. Furthermore, analysis of the dry extract from the fruit reveals a fiber content of 22.3% and mineral elements such as calcium, carbon, nitrogen, magnesium, manganese, iron, copper, zinc, aluminum, potassium, iodine, and selenium. Similarly, the leaves contain 42.4% fiber and elements including carbon, nitrogen, aluminum, copper, iron, zinc, and potassium (Sedaghathoor et al. 2006). Other studies have indicated that both the leaves and fruits of this plant are rich in mineral elements such as potassium, copper, manganese, and iron, along with vitamins A, K, C, and D (Braga et al. 2013; Musavi et al. 2016).

### 3.3 Metabolic Effects of *V. arctostaphylos*

**FIGURE 4:**
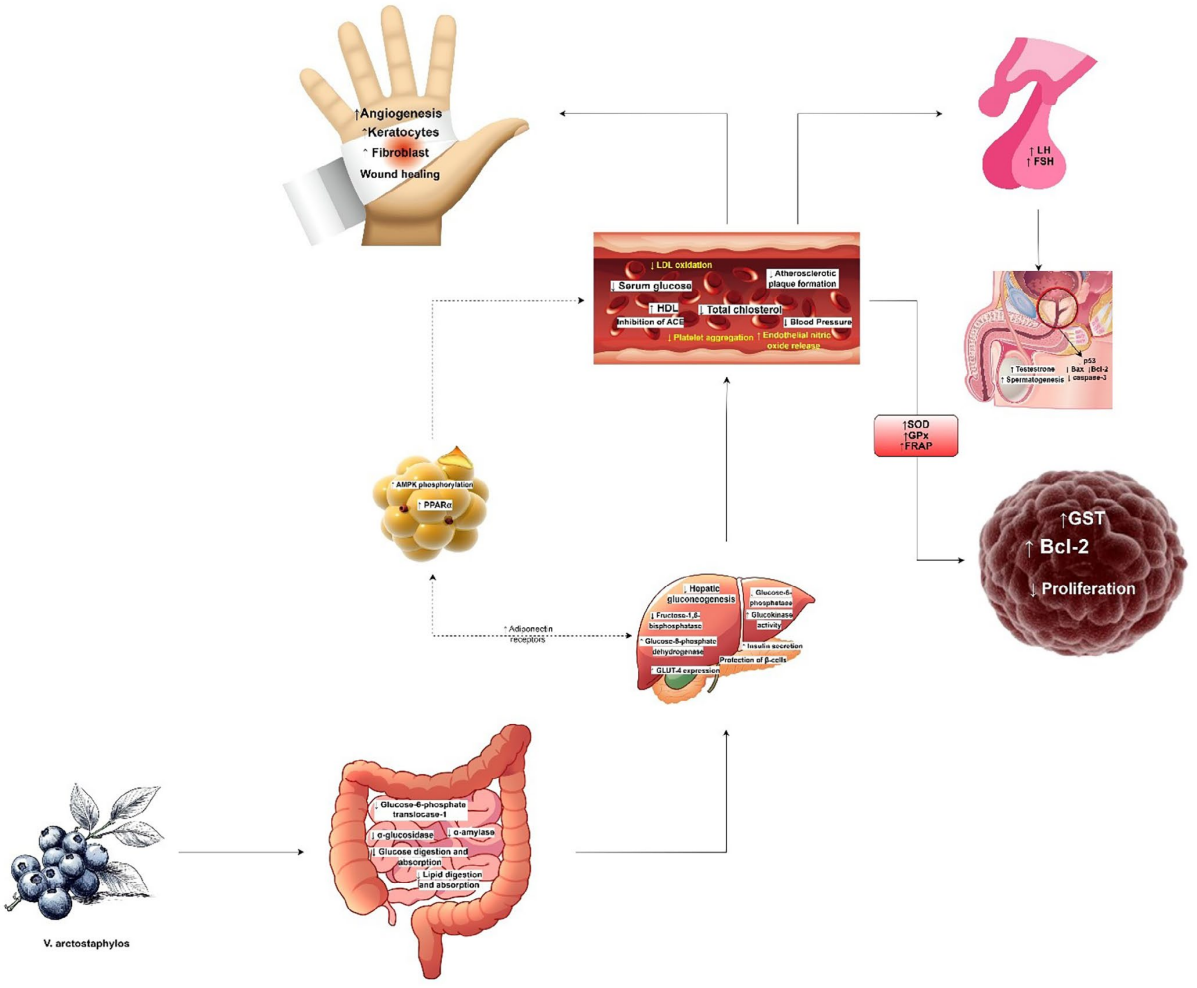
*V. arctostaphylos*, rich in anthocyanins, flavonoids (quercetin, kaempferol, myricetin), and phenolic acids, exhibits diverse therapeutic effects, notably antihypertensive, anti‐obesity, and hypolipidemic activities. Mechanistically, it modulates lipid and glucose metabolism by inhibiting 3‐hydroxy‐3‐methylglutaryl‐coenzyme A reductase (HMG‐CoA reductase), glucose‐6‐phosphatase (G6Pase), angiotensin‐converting enzyme (ACE), α‐glucosidase, α‐amylase, and glucose‐6‐phosphate translocase‐1 (G6PT1), while activating endothelial nitric oxide synthase (eNOS), AMP‐activated protein kinase (AMPK), peroxisome proliferator‐activated receptor alpha (PPARα), and upregulating glucose transporter type 4 (GLUT‐4). These actions improve blood pressure, lipid profiles, and glucose homeostasis. Its anti‐cancer potential stems from increasing glutathione S‐transferase (GST) expression and inhibiting matrix metalloproteinases (MMPs) via tissue inhibitor of metalloproteinases (TIMP) upregulation. Furthermore, antioxidant, anti‐inflammatory, and wound‐healing properties involve modulating growth factor expression and pathways like phosphatidylinositol 3‐kinase/protein kinase B/mammalian target of rapamycin (PI3K/PKB/mTOR) and nuclear factor‐kappa B/mitogen‐activated protein kinase (NF‐κB/MAPK), benefiting cardiovascular health, diabetes, and male reproduction. In male reproductive health, it improves sperm parameters, potentially through antioxidant effects and hypothalamic–pituitary‐gonadal (HPG) axis modulation, with compounds like tribulosin and protodioscin playing a role, alongside protection of spermatogonia via molecular pathways. Finally, its antioxidant capacity is evident through free radical scavenging and enhancing antioxidant enzyme activity, including superoxide dismutase (SOD), catalase (CAT), and glutathione peroxidase (GPx). FRAP (ferric reducing ability of plasma) will increase due to its antioxidant activity.

**FIGURE 5:**
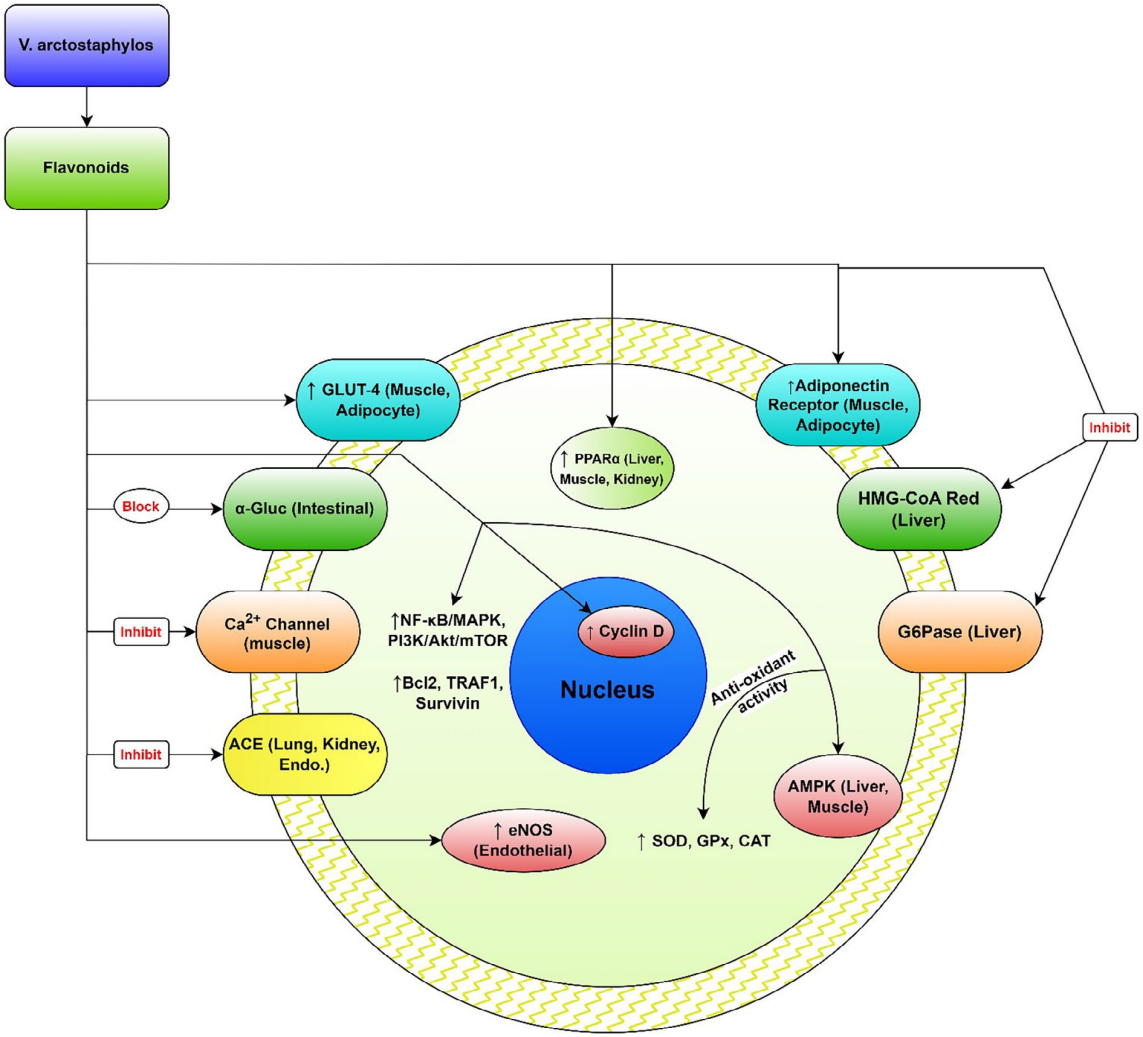
*V. arctostaphylos* fruit extract, rich in bioactive compounds like flavonoids and anthocyanins, exhibits antihypertensive, anti‐obesity, and hypolipidemic effects. These compounds inhibit key enzymes: inhibiting HMG‐CoA reductase (3‐hydroxy‐3‐methylglutaryl‐coenzyme A reductase) to reduce cholesterol; inhibiting glucose‐6‐phosphatase, thereby decreasing hepatic glycogenolysis; inhibiting angiotensin‐converting enzyme (ACE) to help lower blood pressure; and suppressing α‐glucosidase and α‐amylase to reduce glucose absorption. It also activates endothelial nitric oxide synthase (eNOS), promoting vasodilation and contributing to blood pressure reduction. The extract enhances the expression of glucose transporter type 4 (GLUT‐4), improving glucose uptake. Finally, by upregulating AMP‐activated protein kinase (AMPK), peroxisome proliferator‐activated receptor α (PPARα), and adiponectin receptors, it improves lipid metabolism and increases antioxidant enzyme activities such as superoxide dismutase (SOD), glutathione peroxidase (GPx) and catalase (CAT), contributing to the reduction of oxidative stress.

**FIGURE 6:**
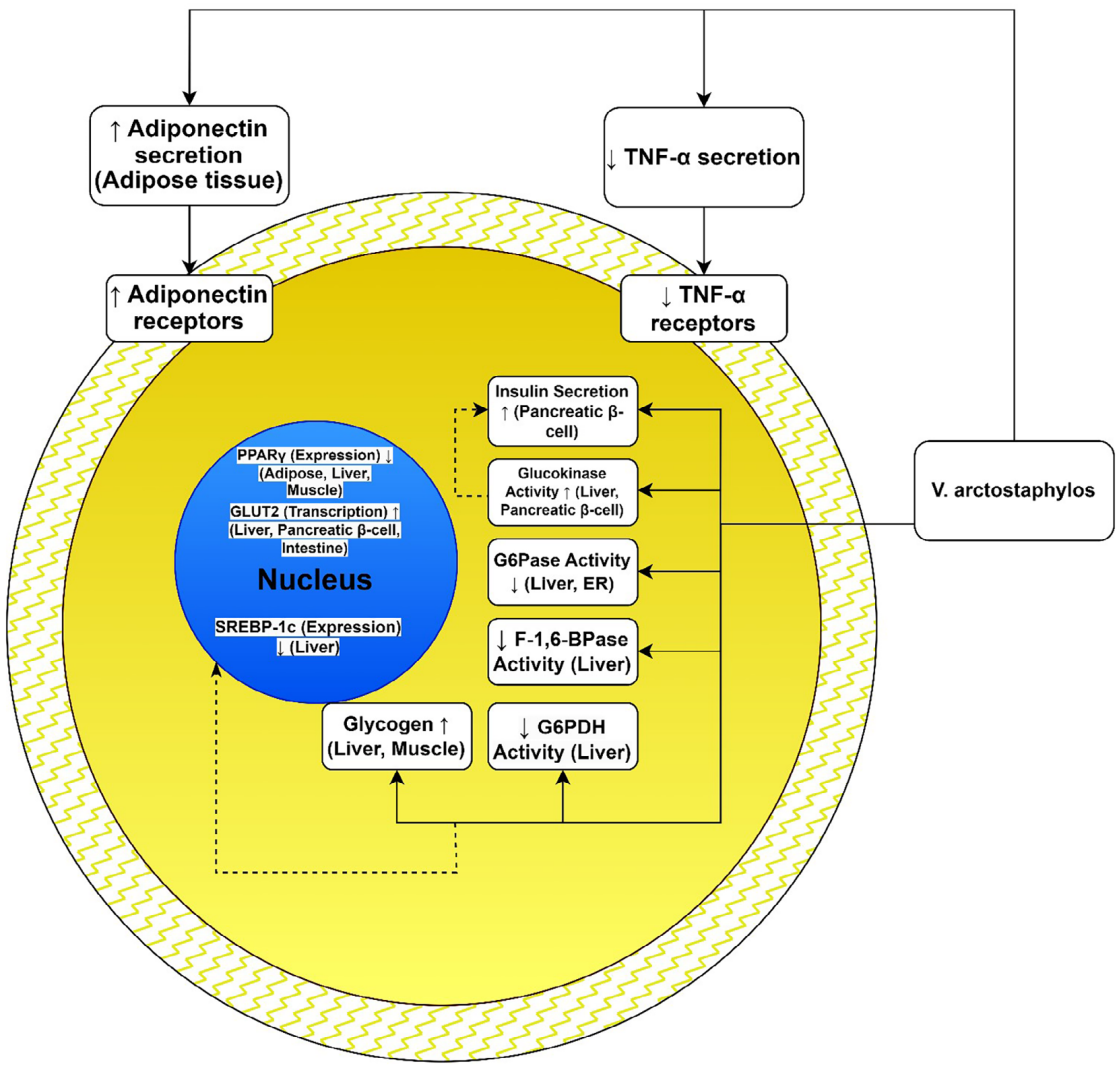
*V. arctostaphylos* fruit extract exhibits promising anti‐diabetic properties, demonstrating both hypoglycaemic and hypolipidemic effects. It modulates key enzymes in glucose metabolism: increasing hepatic GK (glucokinase) and G6PDH (glucose‐6‐phosphate dehydrogenase) activity, simultaneously decreasing G6Pase (glucose‐6‐phosphatase) and FBPase (fructose‐1,6‐bisphosphatase) activity, leading to enhanced glycogen storage and reduced gluconeogenesis. Furthermore, *V. arctostaphylos* improves insulin signaling by upregulating the transcription of IRS1 (insulin receptor substrate 1) and GLUT2 (glucose transporter 2). It influences lipid metabolism by downregulating the expression of PPARγ (peroxisome proliferator‐activated receptor gamma) and SREBP‐1c (sterol regulatory element‐binding protein 1c). The extract increases the expression of miR27‐b, correlating with the improvement of histopathological abnormalities. The extract also increases levels of insulin and adiponectin and decreases the levels of TNF‐α. These findings highlight *V. arctostaphylos* as a potential therapeutic agent with multiple mechanisms of action in diabetes management.

The presence of anthocyanins and various flavonoids in *V. arctostaphylos* contributes to its antihypertensive and antihyperlipidemic effects. Among these flavonoids, flavonols are the predominant chemical structures, known for providing food color and flavor, preventing fat oxidation, and safeguarding vitamins and enzymes (Islam et al. 2022). A randomized, double‐blind, placebo‐controlled clinical trial by Kianbakht et al. (2014) involving 40 hyper‐cholesterolemic and hyper‐triglyceridemic patients demonstrated that consuming 350 mg of *V. arctostaphylos* fruit extract three times daily for 2 months led to increased levels of high‐density lipoprotein cholesterol (HDL‐C) and decreased levels of triglycerides, low‐density lipoprotein cholesterol (LDL‐C), and total cholesterol compared to baseline and the placebo group (Kianbakht et al. 2014). Another randomized, double‐blind, placebo‐controlled clinical trial by Soltani et al. (2014) showed that 500 mg of dried granules during 4 months in hyperlipidemic patients reduced total cholesterol (TC), LDL‐C, TG, and MDA compared to placebo (Soltani et al. 2014) (Table 2; Figure 4). Furthermore, the antihypertriglyceridemic and antihypercholesterolemic effects of *V. arctostaphylos* fruit extract are attributed to flavonoid bioactive compounds such as quercetin, kaempferol, and myricetin, which inhibit 3‐hydroxy‐3‐methylglutaryl‐coenzyme A reductase and glucose‐6‐phosphatase, and up‐regulate adenosine monophosphate‐activated protein kinase phosphorylation, peroxisome proliferator‐activated receptor‐α, and adiponectin receptors (Naveed et al. 2018; Mohtashami et al. 2019). Animal studies also support the efficacy of lower doses of *V. arctostaphylos* in treating hyperlipidemia and hyperglycemia. For instance, Firouzeh (2016) demonstrated that administering 150 mg/kg/day of fruit extract for 30 days effectively controlled streptozotocin‐induced hyperglycemic and hyperlipidemic effects in rats, resulting in decreased serum blood glucose and cholesterol levels, and increased HDL levels and paraoxonase activity compared to the control group (Firouzeh 2016). Similarly, another animal study on alloxan‐diabetic Wistar rats revealed that after 21 days of daily intake of 200 and 400 mg/kg fruit extract, postprandial blood glucose levels, triglycerides, and total cholesterol significantly decreased compared to the control group. Additionally, the study reported significant increases in pancreatic insulin and cardiac GLUT‐4 mRNA expression, as well as antioxidant enzyme activity (superoxide dismutase, glutathione peroxidase, and catalase) in the treated groups (Feshani et al. 2011) (Figure 5). These effects are attributed not only to the inhibition of glucose and intestinal lipid digestion and absorption, and stimulation of pancreatic β‐cells, but also to the antioxidant properties of *V. arctostaphylos*, which protect organs involved in the storage and synthesis of these compounds (Christodoulou et al. 2014; Su et al. 2003). In a randomized, double‐blind, placebo‐controlled clinical trial, 250 mg of *V. arctostaphylos* extract administered twice daily for 8 weeks reduced fasting glucose levels in hyperglycemic type 2 diabetic patients compared to baseline and the placebo group (Abidov et al. 2006). Animal studies conducted on alloxan‐induced diabetic rats demonstrated that doses of 500 and 1000 mg/kg of leaf and fruit extracts of *V. arctostaphylos* for sixty days decreased fasting glucose and HbA1c levels, while increasing serum insulin levels without inducing reno‐hepatotoxicity (Kianbakht and Hajiaghaee 2013). Additionally, an experimental study on the hepatoprotective effect of *V. arctostaphylos* fruit extract in diabetic rats revealed that treatment with this plant fruit extract increased insulin and adiponectin levels, resulting in reduced levels of free fatty acids, reactive oxygen species, and TNF‐α in the serum of diabetic rats (Ravan et al. 2017). Moreover, *V. arctostaphylos* enhanced hepatic glucokinase and glucose‐6‐phosphate dehydrogenase activities, leading to elevated glycogen concentrations, while simultaneously decreasing glucose‐6‐phosphatase and fructose‐1,6‐bisphosphatase activities. The treatment also upregulated insulin receptor substrate 1 and glucose transporter 2 transcription, while downregulating peroxisome proliferator‐activated receptor gamma and sterol regulatory element‐binding protein 1c expression. Additionally, *V. arctostaphylos* notably increased miR27‐b expression in hepatic tissues, significantly normalizing histological abnormalities in diabetic rats compared to the control group. This study highlights the hypoglycemic and hypolipidemic effects of *V. arctostaphylos*, contributing to a more comprehensive understanding of its antidiabetic properties (Saliani et al. 2023; Bharti et al. 2018) (Figure 6).

**TABLE 2:** A review of clinical trials investigating the therapeutic role of *V. arctostaphylos* in lipid profile.

Study (Year)	Registration number	Subjects enrolled	Follow up period	Administration route/dose	Type of disease	Type of study
Kianbakht et al. (2014)	IRCT201112242288N4	105 male and female patients (20–60 years old)	2 months	Orally/one capsule contains 350 mg	Hyperlipidemic patients	Randomized double‐blind placebo‐controlled
Soltani et al. (2014)	—	50 hyperlipidemic adult patients (≥ 18 years old)	4 months	Orally/one capsule contains 500 mg of dried granules	Hyperlipidemic patients	Randomized double‐blind placebo‐controlled
Mohtashami et al. (2019)	IRCT201701282288N11	103 aged between 40 and 80 years old	2 months	Orally/one capsule contains 350 mg	Hypertensive hyperlipidemic type 2 diabetic patients	2‐arm, randomized, double‐blind, placebo‐controlled parallel‐group trial
Asgary et al. 2016	IRCT201507309662N10	40 hyperlipidemic adult patients (≥ 18 years old)	28 days	500 mg/twice daily	Adult hyperlipidemic patients	Randomized, double‐blind, placebo‐controlled
Karlsen et al. 2010	—	62 hyperlipidemic adult patients (≥ 18 years old)	4 Weeks	330 mL juice/day	Adult hyperlipidemic patients	Randomized controlled trial
Basu et al. 2010	—	66 hyperlipidemic adult patients (50.0 ± 6 years old)	8 Weeks	350 g/day	Adult hyperlipidemic patients	Randomized controlled
Abidov et al. 2006	—	44 hyperlipidemic adult patients (46.0 ± 15 years old)	4 Weeks	250 mg/day	Adult hyperlipidemic patients with Type 2 diabetes	Randomized placebo‐controlled

### 3.4 Effects of *V. arctostaphylos* on Cardiovascular System

The cardiovascular effects of the extract from *V. arctostaphylos* are attributed to anthocyanin compounds, including delphinidin, petunidin, and malvidin. Flavonoids, such as kaempferol and quercetin, play a significant role in various plant activities, including regulating cell growth, protecting against environmental factors, and attracting pollinators (Rodríguez De Luna). Kaempferol and quercetin, abundant in onions, apples, and broccoli, protect cardiac cells from mitochondrial damage, reducing cardiovascular complications (Ferreyra and Scalbert A). They reduce LDL oxidation and platelet aggregation, inhibiting the formation of atherosclerotic plaques (Jan et al. 2022). Animal studies on renal hypertensive rats demonstrate that *V. arctostaphylos* extract improves left atrial, pulmonary artery, and mean blood pressure in treatment groups receiving 75 mg/kg of the extract (Khalili et al. 2011). In a randomized, double‐blind, placebo‐controlled clinical trial involving 50 overweight/obese hypertensive patients, capsules containing 400 mg of *V. arctostaphylos* fruit extract, administered three times daily for 3 months, resulted in significant reductions in systolic blood pressure (SBP) from 152.1 ± 7.7 to 140.5 ± 10 and diastolic blood pressure (DBP) from 90.3 ± 8 to 82.1 ± 8.8 compared to baseline and the placebo group (Kianbakht and Hashem‐Dabaghian 2019). Compounds in *V. arctostaphylos* act through angiotensin‐converting enzyme inhibition, endothelial nitric oxide release enhancement, and α1‐antagonism, thereby lowering systolic/diastolic blood pressure (Sakaida et al. 2007). Atherosclerosis, a disorder associated with hyperlipidemia and hypertension, involves chronic inflammatory conditions influenced by various factors such as pro‐inflammatory cytokines and adhesion molecules affecting vascular endothelial cells through paracrine signaling. In a randomized double‐blind, placebo‐controlled clinical trial involving 20 patients with hyperlipidemia (cholesterol and triglyceride > 200), a capsule containing 500 mg twice daily for 28 days of *V. arctostaphylos* fruit extract reduced serum levels of interleukin‐6 (IL‐6), C‐reactive protein (CRP), intercellular adhesion molecule (ICAM‐1), tumor necrosis factor‐α (TNF‐α), and vascular cell adhesion molecule (VCAM‐1), acting as atherogenic biomarkers and reducing the risk of atherosclerosis (Asgary et al. 2016). Another clinical study involving patients at risk of cardiovascular disease (CVD) in a randomized controlled trial demonstrated that consuming 330 mL of *V. arctostaphylos* per day for 28 days in 31 patients resulted in a significant decrease in serum levels of inflammatory factors influencing CVD, such as IL‐6, IL‐15, and CRP, compared with the placebo group (Karlsen et al. 2010). In another similar randomized controlled clinical trial conducted by Basu et al. (2010) on the cardio‐protective effects of *V. arctostaphylos* in 48 patients with metabolic syndrome, consuming 350 g/day for 8 weeks of fresh *V. arctostaphylos* fruit reduced systolic and diastolic blood pressures, plasma oxidized LDL, and malondialdehyde compared with controls (Basu et al. 2010). These results, consistent with other studies, suggest that *V. arctostaphylos* can protect the heart against risk factors for atherosclerotic CVD through various mechanisms (Figure 4).

### 3.5 Anti‐Inflammatory and Antioxidant Effects

Khordad et al. (2024) determined that the LD_50_ of *V. arctostaphylos* is 1.732 g/kg, indicating that doses up to 732 mg/kg of this fruit extract are non‐toxic and suitable for use in animal models. They also found that the total phenolic content (TPC) of this hydroalcoholic extract was 229.4 ± 10.7 mg GAE/g, and the total flavonoid content (TFC) was 120.9 ± 11.7 mg RUE/g. Furthermore, they measured the DPPH free radical scavenging activity at 519.1 ± 10.1 μmol Trolox equivalents per 10 g of dried extract and the ferric reducing antioxidant power (FRAP) content at 1516.6 ± 55.1 mmol Fe^2+^ per mg of dried extract (Khordad et al. 2024). Cinnamic acid and p‐coumaric acid serve as precursors for the flavonoid's pinocembrin and naringenin, respectively. Both flavonoids have garnered significant attention for their antimicrobial, antioxidant, antitumor, and anti‐inflammatory properties (Tungmunnithum et al. 2018). Kaempferol has demonstrated therapeutic effects against cancer and inflammation through various mechanisms of action. This compound acts as a potent scavenger of free radicals and superoxide radicals while preserving the activity of various antioxidant enzymes such as catalase (CAT), glutathione peroxidase (GPx), and Glutathione‐S‐transferase. These effects have shown benefits in various animal disease models, including experimental allergic encephalomyelitis and diabetes (Rajendran 2014). The studies presented explore the antioxidant capabilities of *V. arctostaphylos* in rodents, focusing on enzymes such as GPx, SOD, CAT, FRAP, inducible nitric oxide synthase (iNOS), and other oxidative stress indicators. In a study by Mohebbati et al., they demonstrated the hepatoprotective effects of *V. arctostaphylos* against carbon tetrachloride‐induced acute liver injury in rats, noting increased activity of superoxide dismutase (SOD), CAT, and GPx alongside reduced lipid peroxidation. Similarly, Foroughi et al. (2017) investigated the impact of *V. arctostaphylos* extract on oxidative stress biomarkers in diabetic rat livers, revealing enhanced activity of SOD, CAT, and GPx and decreased malondialdehyde levels, indicative of reduced oxidative stress. Additionally, Yavari et al. (2017) explored the protective effects of *V. arctostaphylos* against oxidative stress in hypercholesterolemic rats, finding increased SOD and CAT activity, reduced lipid peroxidation, and decreased iNOS and TNF‐α levels. These studies collectively highlight the potential of *V. arctostaphylos* in mitigating oxidative stress and modulating antioxidant enzyme activity in various disease states in rodent models. Research on *V. arctostaphylos* demonstrates its therapeutic potential in mitigating tissue injury through modulation of the phosphoinositide 3‐kinase/protein kinase B (PI3K/PKB) pathway and reduction of reactive oxygen species (ROS) in rat and mice models (Akbaribazm et al. 2021; Schink 2018). A study investigated *V. arctostaphylos* protective effects against oxidative stress‐induced liver injury in rats, revealing that this plant fruit extract supplementation suppressed ROS generation, lipid peroxidation, and activated the PI3K/PKB pathway, suggesting hepatoprotective mechanisms (Yan et al. 2016). Another study explored this plant fruit extract's role in mitigating myocardial ischemia–reperfusion injury in mice, indicating that *V. arctostaphylos* administration reduced ROS production, oxidative stress markers, and activated the PI3K/PKB pathway, leading to enhanced myocardial cell survival. These findings underscore the potential therapeutic benefits of *V. arctostaphylos* in combating oxidative stress and tissue injury in both liver and cardiac tissues in experimental models (Kravchenko et al. 2022). The DPPH and FRAP antioxidant tests conducted on one gram of dry fruit samples of *V. arctostaphylos* yielded values ranging from 0.143 to 0.297 mmol and 130.719–346.115 μmol Fe, respectively, indicating the high antioxidant properties of its extracts (Saral et al. 2015). In a study by Hasanloo et al. (2011) on the antioxidant properties of *V. arctostaphylos* fruit native to Iran, the total phenolic content was found to be 42.73 ± 1.5 mg gallic acid equivalents (GAE), and the anthocyanin content was 76 ± 1.00 mg (Hasanloo et al. 2011).

### 3.6 Anticancer Effects of *V. arctostaphylos*

One therapeutic objective in benign prostatic hyperplasia (BPH) is to elevate Glutathione‐S‐transferase (GST) gene expression, which is typically suppressed in early cancer stages. Studies indicate that *V. arctostaphylos*, through its antioxidant and apoptotic properties, upregulates GST gene expression by mitigating DNA hyper‐methylation and restraining the proliferation of prostate cancer cell line (PC‐3) (Gorbanzadeh and Zaefizadeh 2017). Matrix metalloproteinases (MMPs) facilitate tumor cell invasion and metastasis by degrading extracellular matrix proteins like collagen and laminin. Tissue inhibitor of metalloproteinase (TIMP) counters MMP activity, inhibiting metastasis. *V. arctostaphylos* extract, by upregulating TIMP‐1 and TIMP‐2, inhibits MMP‐2 and MMP‐9, impeding gastric cancer cell survival and metastasis (Norouz‐zadeh et al. 2021). Research on the effects of *V. arctostaphylos* fruit extract on various cancer cell lines, including breast cancer (MCF‐7, MDA‐MB‐231, 4 T1), colon cancer, glioblastoma, and others, reveals its potential in cancer therapy. Studies indicate that *V. arctostaphylos* extract exhibits antiproliferative and apoptotic effects on MCF‐7 breast cancer cells, while it may also inhibit cell proliferation and induce apoptosis in MDA‐MB‐231 triple‐negative breast cancer cells. However, limited studies specifically focusing on the effects of *V. arctostaphylos* extract on 4 T1 breast cancer cells are available. Research suggests that *V. arctostaphylos* extract may exert anticancer effects on various cancer cell lines, including prostate cancer (PC‐3), gastric cancer (AGS, MKN‐45), leukemia (HL60), among others (Gorbanzadeh and Zaefizadeh 2017). These studies indicate that *V. arctostaphylos* extract may induce apoptosis, inhibit proliferation, and modulate signaling pathways associated with cancer progression, including PI3K/PKB and MAPK pathways. While promising, further in‐depth studies focusing on specific cancer cell lines are warranted to elucidate the mechanisms of action and therapeutic efficacy of *V. arctostaphylos* extract comprehensively. Multidrug resistance (MDR) often undermines chemotherapy effectiveness in cancer treatment, with ATP‐binding cassettes (ABCs) like MRP‐1 facilitating drug efflux. *V. arctostaphylos* extract enhances chemotherapy efficacy on gastric cancer cell lines (AGS and MKN‐45) by suppressing MRP‐1 expression, curtailing their growth and viability (Esmaeili et al. 2021). Moreover, comparative studies on *V. arctostaphylos* and *V. myrtillus* extracts on human tumor cell lines HL60 and НСТ116 reveal their anthocyanin‐rich compositions induce apoptosis and restrain tumor cell proliferation (Karcheva‐Bahchevanska et al. 2017).

### 3.7 Wound Healing Properties *V. arctostaphylos*

Wound healing encompasses a complex interplay of inflammatory, antioxidant, antiseptic, and angiogenesis processes, targeting pathways involved in coagulation, homeostasis, inflammation, proliferation, and wound remodeling phases. Effective treatment strategies aim to address all stages of wound healing. *V. arctostaphylos* extract harbors compounds with angiogenic, anti‐inflammatory, and antioxidant properties, alongside those facilitating the proliferation and differentiation of dermal and epidermal keratinocytes, melanocytes, fibroblasts, and dermal lymphatic endothelial cells, making it a promising candidate for wound healing applications. Research by Tayari et al. (2014) demonstrated enhanced wound healing in Wistar rats treated with an ointment containing 20% *V. arctostaphylos* extract over 21 days, surpassing both control and zinc oxide ointment groups, highlighting the potential therapeutic efficacy of *V. arctostaphylos* extract in wound management (Figure 4).

### 3.8 Effects on Male Reproductive System

ROS play a crucial role in intracellular signaling, sperm capacity, acrosome response, and spermatozoon‐oocyte fusion. Spermatogonia and sperm DNA and membrane's unsaturated fatty acids are strongly affected by free radicals. After oxidative‐reducing reactions, they lose their integrity, leading to cellular necrosis. As a result, the morphology, motility, and viability of spermatozoa are altered, reducing the number of active spermatozoa capable of fertilization. *V. arctostaphylos* has been shown to have antioxidant and anti‐inflammatory effects. In an experimental study, *V. arctostaphylos* decreased testosterone serum levels, increased LH and FSH levels, improved stereological and sperm parameters, down‐regulated the p53, caspase‐3, and Bax genes, and up‐regulated Bcl‐2 gene expression (Akbaribazm et al. 2024). Additionally, this treatment decreased serum levels of NO and increased testis tissue FRAP and MDA levels compared with the control group. In another study, Akbari Bazm et al. (2020) demonstrated in a mouse model that 200 and 400 mg/kg of *V. arctostaphylos* extract can enhance the hypothalamus‐pituitary‐gonad axis, exhibiting antioxidant and anti‐inflammatory effects. Their findings suggest that the aphrodisiac compounds in *V. arctostaphylos* likely enhance testosterone synthesis and regulate the secretion of luteinizing hormone and follicle‐stimulating hormone. Additionally, they reported that *V. arctostaphylos* can protect the spermatogenic lineage in individuals exposed to anabolic androgenic steroids (AAS), such as oxymetholone, by inhibiting mitochondrial apoptosis pathways (Akbari Bazm et al. 2020). In a histopathological and morphometric evaluation by Ghaffarian et al. (2023) in an animal model on rats under cisplatin toxicity, doses of 100 and 200 mg/kg of *V. arctostaphylos* improved the thickness of the germinal epithelium and protected spermatogonial cells from damage (Ghaffarian et al. 2023) (Figure 4).

### 3.9 Antibacterial Effects

*V. arctostaphylos* contains proanthocyanidins that inhibit bacterial adhesion and subsequent proliferation. While anthocyanins are common in fruits similar to *V. arctostaphylos*, research has shown that the anthocyanidin compounds specific to this fruit have unique antibacterial properties (Khodadadi et al. 2021). In traditional medicine, various plants like lemon balm ( *Melissa officinalis* L.), garlic ( *Allium sativum* L.), tea tree (*Melaleuca alternifolia* L.), bearberry ( *Arctostaphylos uva‐ursi* L.), and cranberry juice ( *Vaccinium macrocarpon* L.) are utilized for their broad antibacterial properties in treating urinary infections (Rios and Recio 2005; Joshi 2018; Islam et al. 2022). Cowan's review article also highlights the antimicrobial potential of plant materials, including fruits such as cranberries and blueberries, against bacteria like *Escherichia coli* (Cowan 1999). Khodadadi et al. demonstrated that *V. arctostaphylos*, or lingonberry, can effectively inhibit the adhesion and proliferation of bacteria such as *E. coli* and *Helicobacter pylori* due to its proanthocyanins' ability to block bacterial adhesion to host cells, thus preventing colonization and infection (Khodadadi et al. 2021). Additionally, in her review study, Katarzyna Kowalska mentioned the potential of Lingonberry ( *Vaccinium vitis‐idaea* L.) fruit in inhibiting bacteria growth, biofilm formation, visible plaque index, and fungi growth (Kowalska 2021). Teimouri (2015) evaluated the antibacterial activities of essential oil extracted from the flowering shoots of *V. arctostaphylos* against various bacteria in vitro. The study indicated that these extracts exhibited moderate antibacterial activities against bacteria such as *E. coli* , *E. faecalis* , *K. pneumoniae* , and *S. pneumoniae* (Teymouri 2014) (Tables 2 and 3).

**TABLE 3:** The predominant anthocyanins, flavonols and polyphenols of the *V. arctostaphylos* plant.

References	Dosage used/duration	Plant part	Animal model	Observed effects
Firouzeh 2016	150 mg/kg/30 days	Fruit	Rats (Wistar)	Decreased the serum blood glucose, cholesterol and HDL levels
Feshani et al. 2011	Of 200 and 400 mg/kg/21 days	Fruit	Rat (Wistar)	The level of antioxidant enzyme activity (superoxide dismutase, glutathione peroxidase, and catalase) were significantly increased in the groups treated with the extract
Khalili et al. 2011	75 mg/kg/28 days	Leaves	Rat (Wistar)	Improved left atrial, pulmonary artery and mean blood pressure
Saliani et al. 2023	400 mg/kg/42 days	Fruit	Wistar rats	VAE remarkably enhanced the expression of miR27‐b in the hepatic tissues of diabetic rat

## 4 Conclusion

This review covers the ethnomedical applications, phytochemical composition, and pharmacological properties of various extracts from different parts of *V. arctostaphylos*, with a particular focus on the aerial parts and fruits of this medicinal plant. In Iranian traditional medicine, *V. arctostaphylos* has been used both as a spice and a remedy for numerous ailments. Scientific investigations have concentrated on the fruits of this herb due to their well‐documented use in Iranian folklore. These studies provide evidence supporting its ethnopharmacological applications, indicating that *V. arctostaphylos* is a valuable source for medicinal purposes. Most of the traditional uses of *V. arctostaphylos* have only been validated through in vivo studies. Consequently, human clinical trials are necessary to establish the biological relevance of *V. arctostaphylos* for various diseases and to substantiate its traditional uses accurately. Data on the toxicological properties of *V. arctostaphylos* is limited, highlighting the need for further evaluation of the plant's toxicity and safety. The active compounds of this plant should be purified and assessed for potential drug discovery. Additionally, the mechanisms underlying many of its pharmacological effects remain unknown, necessitating further tests to identify these effects and their molecular targets. Moreover, more research is required to standardize the optimal dosage for the claimed effects of *V. arctostaphylos* in traditional medicine.

## Author Contributions

**Hesam Bighamanganeh:** software (equal), validation (equal), visualization (equal), writing – original draft (equal), writing – review and editing (equal). **Maryam Salabati:** software (equal), supervision (equal), validation (equal), visualization (equal), writing – original draft (equal), writing – review and editing (equal). **Abbas Khalilpour:** data curation (equal), formal analysis (equal), resources (equal), writing – original draft (equal), writing – review and editing (equal). **Mohsen Akbaribazm:** conceptualization (equal), data curation (equal), formal analysis (equal), methodology (equal), resources (equal), validation (equal), visualization (equal), writing – original draft (equal), writing – review and editing (equal). **Nabi Akbarnezhad:** conceptualization (equal), data curation (equal), validation (equal), writing – original draft (equal), writing – review and editing (equal).

## Ethics Statement

The authors have nothing to report.

## Conflicts of Interest

The authors declare no conflicts of interest.

## Data Availability

All data generated or analyzed during this study are included in this published article.
